# Hematopoietic Cells from Pluripotent Stem Cells: Hope and Promise for the Treatment of Inherited Blood Disorders

**DOI:** 10.3390/cells11030557

**Published:** 2022-02-05

**Authors:** Ilaria Rao, Laura Crisafulli, Marianna Paulis, Francesca Ficara

**Affiliations:** 1 IRCCS Humanitas Research Hospital, Via Manzoni 56, 20089 Rozzano, Italy; ilaria.rao@humanitasresearch.it (I.R.); laura.crisafulli@humanitasresearch.it (L.C.); marianna.paulis@humanitasresearch.it (M.P.); 2 Department of Biomedical Sciences, Humanitas University, Via Rita Levi Montalcini 4, 20090 Pieve Emanuele, Italy; 3 UOS Milan Unit, Istituto di Ricerca Genetica e Biomedica (IRGB), CNR, 20138 Milan, Italy

**Keywords:** pluripotent stem cell, iPSC, hematopoietic stem cell, hematopoiesis, differentiation, inherited blood disorders, gene therapy, gene editing, genomic disorders, chromosome transplantation

## Abstract

Inherited blood disorders comprise a large spectrum of diseases due to germline mutations in genes with key function in the hematopoietic system; they include immunodeficiencies, anemia or metabolic diseases. For most of them the only curative treatment is bone marrow transplantation, a procedure associated to severe complications; other therapies include red blood cell and platelet transfusions, which are dependent on donor availability. An alternative option is gene therapy, in which the wild-type form of the mutated gene is delivered into autologous hematopoietic stem cells using viral vectors. A more recent therapeutic perspective is gene correction through CRISPR/Cas9-mediated gene editing, that overcomes safety concerns due to insertional mutagenesis and allows correction of base substitutions in large size genes difficult to incorporate into vectors. However, applying this technique to genomic disorders caused by large gene deletions is challenging. Chromosomal transplantation has been proposed as a solution, using a universal source of wild-type chromosomes as donor, and induced pluripotent stem cells (iPSCs) as acceptor. One of the obstacles to be addressed for translating PSC research into clinical practice is the still unsatisfactory differentiation into transplantable hematopoietic stem or mature cells. We provide an overview of the recent progresses in this field and discuss challenges and potential of iPSC-based therapies for the treatment of inherited blood disorders.

## 1. Introduction 

Hereditary blood disorders comprise some 5000 distinct diseases; they are caused by alterations in more than 200 genes that modify the function and/or the number of different blood elements, namely red blood cells (RBCs), white blood cells, and platelets. Most inherited blood disorders are due to point mutations affecting hematopoietic stem cells (HSCs) or their committed progeny, leading to hematopoiesis or cell lineage-specific defects. For example, more than 300 different mutations on the β-globin gene (HBB) are responsible for β-hemoglobinopathies [1,2], diseases due to defective RBCs. In other inherited disorders, more complex genomic alterations can cause a loss or a gain of a specific function. Chromosomal mutations, which include gross deletions, large insertions, duplications, complex rearrangements, and gene fusions, can have an impact of gene dosage resulting in reduced, increased, or ectopic gene expression [3].

Patients affected by hereditary blood disordered often need RBC or platelet transfusions, a procedure linked to the availability of compatible donors and that requires to be repeated. Allogeneic HSC transplantation is the only definitive cure for most blood genetic disorders. However, it is limited by the availability of HLA matching donors, and it can be associated with significant risks such as graft failure, graft versus host disease (GVHD), delayed immune reconstitution, and, in some case, risk of disease recurrence [4]. Therefore, to cure this type of disorders, there are currently many efforts towards developing safe and effective ex-vivo gene therapy (GT) approaches, such as autologous transplantation strategies based on genetically corrected HSCs [5].

Cellular and gene therapies for hematologic disorders can greatly benefit from the prompt availability of hematopoietic stem/progenitor cells (HSPCs) in the bone marrow (BM), in the cord blood and in mobilized peripheral blood (PB). Another promising option is the generation of HSCs or mature blood cells from induced pluripotent stem cells (iPSCs) or embryonic stem cells (ESCs) [6,7,8]. The advantage of iPSCs is that their cell source is easily accessible, that they can be produced at a relatively reasonable price, and that they represent a virtually unlimited source of HSPCs and mature blood cells. Moreover, iPSCs can be autologous, thus preventing rejection and rising fewer ethical issues regarding their collection when compared to ESCs.

With autologous cells, a further step of gene correction is required in patients with genetic mutations. Several different approaches have been considered to address the correction of the genetic defect. Over the past few years, the emergence of new genome editing technology such as clustered regularly interspaced short palindromic repeat (CRISPR)/Cas9 has opened the opportunity to correct genetic mutation in an efficient and precise manner. The use of genome editing tools and iPSC technology for targeting monogenic blood diseases has been recently described [9,10,11,12,13,14,15,16,17,18,19]. Although CRISPR technology coupled to iPSCs represents one of the most promising approaches to gene correction and therapy at present, additional hurdles must be overcome. Among these, the mutation load of iPSCs, which undergo several stresses and long culture periods [20], and the possibility of “off target” effects must be taken into consideration [21]. These limits apply to essentially any genetic disease to be studied. However, some genetic defects, such as “genomic” or “chromosomal” disorders, present a further difficulty since they cannot be treated with conventional techniques. They consist in “structural” abnormalities, which include large deletions or inversions, copy number variations, aneuploidies and complex rearrangements. We proposed a novel approach called chromosomal transplantation (CT) as a solution, using a universal source of wild-type (WT) chromosomes as donor, and iPSCs as acceptor [22,23].

Possible approaches to overcome the correction step would be the generation of “universal” non-immunogenic iPSC lines, or the generation of iPSC biobanks, for HLA-matched allogeneic cell therapy [24,25].

Finally, the ability to recapitulate in vitro the normal pathways of differentiation from pluripotent to multipotent progenitors and mature cells is a prerequisite to obtain functional corrected cells in the desired lineage, including blood cells [7,26].

In this review, an overview of applications of iPSCs for hematological diseases, genome correction approaches, differentiation strategies and future perspectives will be discussed. Although only corrected HSCs will provide stable and definitive cure of inherited blood disorders, we will also discuss benefits from the generation of iPSC-derived mature hematopoietic cells for the treatment of these diseases.

## 2. Genetic and Genomic Hematologic Diseases

Mutations are conventionally classified in two classes, single gene mutations that involve single genes, and chromosomal mutations that involve chromosomal segments containing large portion of one gene, many genes, or whole chromosomes. When these kinds of mutations occur in germ-cell lineages, they cause an inherited disease, or pre-disposition to disease [27], including blood disorders. Moreover, a single disease entity may be the result of any one of several different alterations within the gene responsible for the disease. Few examples of hereditary blood disorders caused by both single gene mutations and chromosomal mutations are mentioned below.

Chronic granulomatous disease (CGD) is an inherited primary immunodeficiency characterized by severe, recurring infections due to absent or poor activity of phagocyte NADPH oxidase. The X-linked form of the disease is caused by mutations in the CYBB gene, which encodes the 91-kD glycoprotein component (gp91-phox) of the NADPH oxidase complex responsible for reactive oxygen species production in phagocytes. The type of mutations identified in patients are largely heterogeneous and include frameshift (20%), nonsense (20%), missense (20%), splice-region mutations (15–20%), but also large and small deletions (10%) and regulatory-region mutations (<5%) [28].

DiGeorge syndrome and velocardiofacial syndrome also known as 22q11.2 deletion syndrome (22q11DS) is a common autosomic dominant genetic disease characterized by broad phenotypic variability. Most patients have a large deletion of 3 Mb in region 11 of the long arm of chromosome 22 (22q11.2del), leading to a haploinsufficiency of more than 100 genes, and comprising coding RNAs, noncoding RNAs, and pseudogenes [29]. Immunodeficiency and hematological alterations such as thrombocytopenia and large platelets observed in 22q11DS are associated to this disease [30].

Fanconi anemia (FA) is a rare genetic disorder characterized by chromosomal instability, variable congenital anomalies, progressive BM failure, and cancer susceptibility [31]. FA patients have progressive loss of HSCs in their BM [32], which clinically manifests as reduced mature cells. Mutations in at least 22 genes, all involved in DNA repair and genome stability, termed complementation groups FANCA-FANCW, have been associated to this disease [33]. All the genes are inherited in an autosomal recessive manner, except for FANCB that is localized on the X chromosome and causes X-linked FA. Molecular diagnosis of FA is complex, not only for the high number of genes associated with its development, but also for the heterogeneity of the mutations, which include large deletions or duplications [34,35].

X-linked agammaglobulinemia (XLA) is mainly caused by a mutation of the Bruton’s tyrosine kinase (*BTK*) gene [36]. XLA patients are characterized by a reduction or lack of mature B lymphocytes, resulting in recurrent bacterial infections in affected males in the first two years of life. The *BTK* gene is located on the long arm of the X chromosome (Xq22.1) and contains 19 exons. Since the gene was discovered, a total of 1815 different *BTK* gene mutations were reported (https://databases.lovd.nl/shared/genes/BTK, accessed on 17 December 2021). They are uniformly distributed throughout the gene and include a huge variety of alterations ranging from point mutations, the most frequent, to large gene rearrangements. Moreover, 3–5% of individuals have a large deletion that extends through the closely linked Translocase of Inner Mitochondrial Membrane 8 (*TIMM8A*) gene [37], a translocase involved in the import and insertion of hydrophobic membrane proteins from the cytoplasm into the mitochondrial inner membrane. The extent of this genetic defect encompassing more than one gene may partially explain the heterogeneity and different severity typical of this hematological disorder. Several different approaches have been considered to address the correction of different genetic mutations.

## 3. Innovative Cell Therapy Approaches for Inherited Blood Disorders

The standard therapy for many patients with inherited blood diseases is HSC transplantation (HSCT), which replaces mutated blood cells and guarantees a definitive cure. The major challenge of HSCT is the availability of appropriate and sufficient donor cells for robust and durable hematopoietic repopulation. The cell source can be either from healthy donors, as in allogeneic HSCT, or patients’ own stem cells, as in autologous HSCT. Compared to allogeneic, autologous transplants present lower risk of complications such as GVHD, graft failure and infections. However, autologous HSCT requires gene modification prior to transplantation to correct gene-causing mutations. This can be achieved through classical GT or through CRISPR/Cas9-mediated gene editing of autologous HSCs. Novel proposed strategies are instead based on iPSCs.

**Figure 1:**
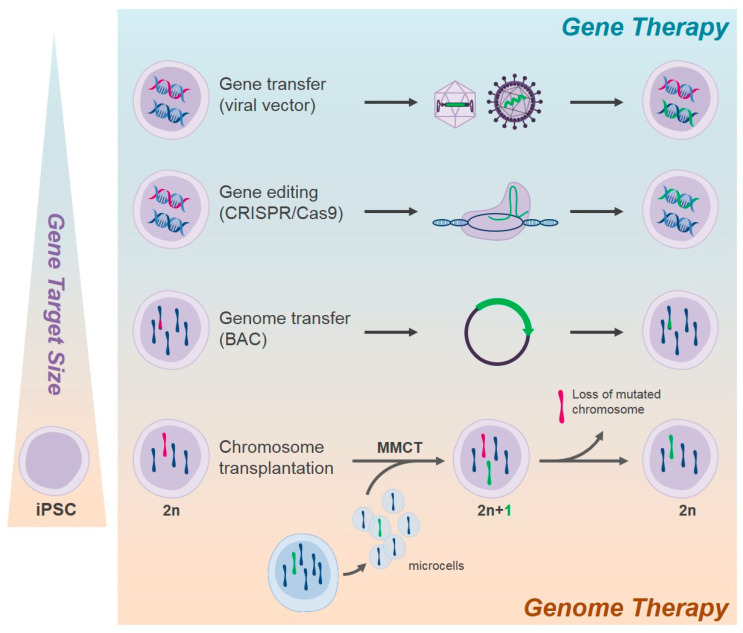
Gene/genome therapy options for the cure of hereditary blood disorders based on gene target size. With gene transfer WT form of the disease gene is added in the genome of the recipient cells; with gene editing the mutated gene is corrected; with BAC-mediated genome transfer the mutated gene including upstream regulatory sequences and intronic regions is substituted with homologous recombination; with chromosome transplantation the entire chromosome containing the altered gene(s) is replaced with a WT chromosome. MMCT, microcell-mediated chromosome transfer.

For hereditary blood disorders, the choice of ex-vivo GT vs. correction of autologous iPSCs followed by hematopoietic differentiation might be dictated by disease type, availability of patient’s own primary HSCs, and nature of the mutation. Compared to primary HSCs, a higher range of correction options are available for iPSCs, and the choice will be subordinated to the size of the gene defect (Figure 1).

Viral-mediated gene transfer or CRISPR/Cas9-mediated gene editing might be ideal as GT platforms for small-size gene defects, while BAC-mediated or CT-mediated genome editing might be the best alternative for gross mutations and chromosomal disorders.

### 3.1. Pluripotent Stem Cells

Pluripotent stem cells (PSCs) can generate all body tissues. The prototypical PSCs are ESCs, obtained from pre-implantation embryos. PSCs that are derived from somatic cells are called induced PSCs (iPSCs); they are relatively more accessible than ESCs and present advantages such as the possibility to generate autologous cells belonging to the desired tissue. Discovery and development of iPSCs have been extensively reviewed elsewhere [38]. Briefly, in 2006 Yamanaka et al. described the possibility to reprogram differentiated murine embryonic fibroblasts into iPSCs in culture following viral-mediated transduction of a cocktail of four main transcriptor factors, namely Oct3/4, Sox2, c-Myc and Klf4 [39]. The following year the same team generated iPSCs from human somatic cells with the same technique [40]. Essentially, ectopic expression of these transcription factors silences the somatic gene expression and activate pluripotency regulatory networks. In the last 15 years, the original procedure for dedifferentiation of somatic cells into iPSCs was improved and many methods are now used to obtain iPSCs from different cell types belonging to different species. Methods for obtaining iPSCs vary in the cocktail of reprogramming factors, way of delivery them, type of somatic cell to be reprogrammed, culture conditions, to name a few, and greatly affect reprogramming efficiency and safety of the final product. In particular, different reprogramming methods have been employed to generate human iPSCs without genome-integrating DNA elements. These methods use, for example, episomal vectors, adenoviral vectors, Sendai viral vectors, plasmids, synthetic mRNA, miRNA, protein transduction and small molecules [41]. This allowed the achievement of a better safety profile by minimizing genomic instability and tumorigenic potential. Importantly, since iPSCs can be generated from adult tissues, each individual could in principle have their autologous iPSC line. Thanks to these features, iPSCs hold a huge potential and are a great promise for regenerative medicine.

### 3.2. Gene Therapy of Pluripotent Stem Cells

Early studies using patient-specific iPSCs as a potential source for autologous cell-based therapy relied on the use of low-efficiency homologous recombination or lentiviral GT [42,43]. A limit of the lentiviral GT system is random integration of the exogenous WT gene into the genome, which can result in undesired mutations. The first proof of principle of the use of iPSCs for GT purposes was provided with sickle cell anemia [44], followed by several other examples. For non-hematological diseases, iPSCs for autologous cell transplantation therapy is already successfully employed in clinical trials [45]. Before the CRISPR/Cas9 era, the rationale behind applying GT for the cure of inherited blood disorders by genetic modification of iPSCs was that only PSCs offered the possibility to perform homologous recombination, to avoid the risks of insertional mutagenesis associated with retroviral transduction. With homologous recombination it is possible to restore the integrity of the entire gene locus, and PSCs offer the opportunity to select, expand and differentiate the most appropriate clone. Our group demonstrated the potential of this approach using a murine model of autosomal recessive osteopetrosis (ARO) carrying a homozygous mutation in T cell immune regulator 1 (*Tcirg1*), the most frequently mutated gene in this disease, which encodes a subunit of the V-ATPase proton channel. We generated iPSCs from a *Tcirg1*^−/−^ ARO mouse and performed BAC-mediated homologous recombination using the full-length WT gene, thus including all endogenous regulatory regions. Corrected iPSCs were then differentiated into early myelo-erythroid progenitors and finally into monocyte-derived funtional, bone resorbing osteoclasts [46]. A similar BAC-mediated homologous recombination approach was shown later to correct the mutated CYBB gene from a CGD patient [47]. Examples of site-specific correction of iPSCs followed by hematopoietic differentiation are TALEN-mediated gene editing of mutated IL-2Rγ gene from a SCID-X1 patient [12], or CRISPR/Cas9-mediated gene targeting of JAK3 to model SCID due mutated JAK3 [48], while transgene CGD correction has been proposed through zinc finger nuclease (ZFN) mediated AAVS1 safe harbor minigene targeting [49]. A major advancement in the field might be the possibility of simultaneously editing and reprogramming autologous fibroblasts employing a CRISPR/Cas9 adenine base editor (ABE), as recently shown [50]. This method reduced cell culture time (human iPSC lines with the desired correction were obtained in 5 weeks) and improved safety since ABE does not induce DNA double-strand brakes and has inferior risk of off-target events.

With the CRISPR/Cas9 technology, the possibility to perform ex-vivo targeted gene correction in primary CD34^+^ cells became a reality [51,52], and many laboratories are actively working to improve efficiency and safety of this technique to bring it to the clinic [53]. Clinical trials for the treatment of sickle cell disease and β-thalassemia are indeed ongoing (ClinicalTrials.gov #NCT03745287 and #NCT03655678). However, generation of HSCs from corrected autologous iPSCs might still be considered as the only alternative to allogeneic HSCT for the cure of hematological diseases in which patient’s own stem cells are either difficult to obtain in sufficient number, or for which classical GT approaches or gene editing are not feasible, such as in case of disorders due to deletion of large portions of the genome, as mentioned earlier. Examples of the first case are diseases characterized by BM aplasia, such as Fanconi anemia (FA), or fibrosis, as in osteopetrosis. FA is a rare X-linked genetic disease caused by germline mutations in any of several genes, including FANCA and FANCB, whose products are involved in DNA repair mechanisms [32]. In FA a progressive HSPC depletion due to cell cycle alterations and elevated cell death leads to BM failure with reduced blood cell formation and severe anemia, and collection of cells for GT purposes is challenging if not performed at very young age before HSC exhaustion [54]. FA fibroblasts and FA fibroblast-derived iPSCs have been recently corrected through the CRISPR/Cas9 technology [55,56]; corrected iPSCs were differentiated into cells with characteristics of definitive hematopoiesis, providing a proof of principle of the feasability of an iPSC-based GT approach for this disease. A further example is osteopetrosis; in ARO patients, BM harvest cannot be performed due to severe BM fibrosis, lack of BM cavity and susceptibility to bone fractures. Since most patients have high frequencies of circulating CD34^+^ cells, the use of HSPC ex vivo expansion protocols coupled to GT has been proposed to overcome these issues [57]. An alternative option might be generation of gene-targeted autologous iPSCs as an unlimited source for corrected HSC production.

A possible approach to treat hematological disorders due to chromosomal mutations could be chromosome transplantation (CT). CT, differently than chromosome transfer, which consists of adding a surplus chromosome, is based on the complete replacement of the defective chromosome with a normal donor one (Figure 1), obtaining the total restoration of a correct normal diploid karyotype (46, XY/XX). Pluripotent stem cells represent an adaptable platform for CT offering the possibility to differentiate into specific lineage of interest the corrected cells. We used this type of approach on human and murine pluripotent stem cells to address treatment of different type of diseases [20,21], including hematological disorders. In detail, we replaced the defective X chromosome in iPSCs derived from a murine model of CGD with a WT X chromosome obtained from a healthy donor [58]. The endogenous defective X chromosome was previously engineered through the CRISPR/Cas9 system to obtain Hprt inactivation, as a mean to discriminate the exogenous X chromosome from the endogenous defective one. In this study we replaced the X chromosome since CGD is a X-linked disorders, but theoretically it is possible to adapt this protocol for many more diseases based on different mutations in different chromosomes.

All genetic diseases with a wide spectrum of mutations in the same gene/chromosome will greatly benefit from a unique GT approach like the substitution of the entire chromosome instead of different GT approaches specific for each type of mutation.

### 3.3. iPSC Biobanks

The implementation of iPSC biobanks would represent a major step forward toward the success of iPSC-based regenerative therapies. “Universal” non-immunogenic iPSC lines can be generated by genetic inactivation of HLAs and overexpression of specific cell surface markers, such as CD47, to mask iPSC-derived cells from the immune system [22]. In addition, somatic cells from healthy donors with known HLA and genome characteristics have been already reprogrammed to initiate creation of iPSC collections. These biobanks are meant to include most HLA subtypes and would be useful to overcome part of the major concerns regarding the use of clinical-grade iPSCs and facilities variability [24,25]. More in general, to translate iPSC-derived HSCs into clinical applications, cells must be obtained following Good Manufacturing Practice (GMP)-grade procedures with increased production costs, which can be demanding for individual centers.

## 4. Strategies to Generate Hematopoietic Stem and Progenitor Cells from Pluripotent Stem Cells

For GT purposes, genetically corrected autologous iPSCs must be differentiated to hematopoietic cells. The goal is to generate transplantable HSCs able to engraft and reconstitute in vivo the entire hematopoietic system. Generation of terminally differentiated cells or committed, non-self-renewing progenitors, would only determine temporary benefits, if any, although here are some isolated exceptions to this general rule (see below, paragraph 5).

**Figure 2:**
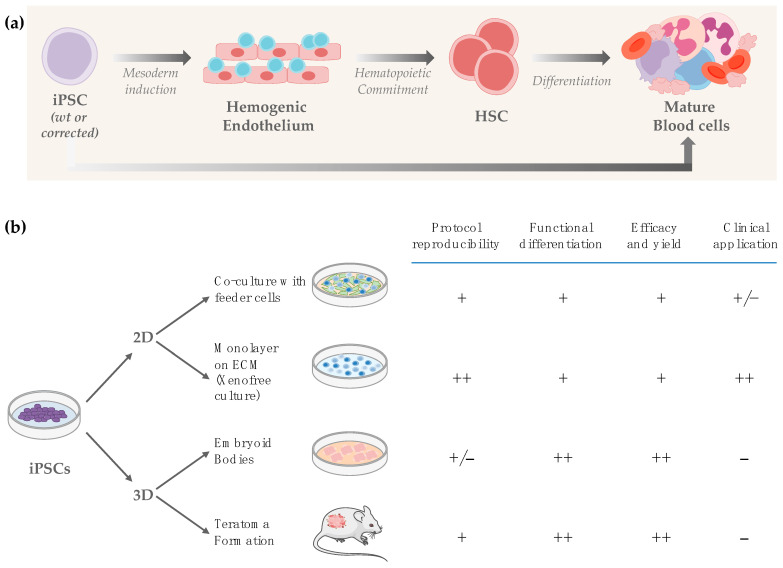
Hematopoietic differentiation from iPSCs. (**a**) Steps to generate blood cells from iPSCs. The HSC stage occurs during definitive hematopoiesis; (**b**) Strategies to generate blood cells from iPSCs. Symbols indicates absence (−), low (+/−), moderate (+), or strong (++) compliance with the highlighted features (protocol reproducibility, functional differentiation, efficacy, yield and clinical application).

Protocols to generate HSCs from PSCs have been developed based on the knowledge of embryonic development. During embryonic development hematopoiesis occurs in temporally and spatially separated waves, named primitive and definitive. Primitive hematopoiesis mainly arises in yolk sac blood islands and in placenta to transiently generate nucleated RBCs, megakaryocytes (Mk) and monocyte-macrophages. Definitive hematopoiesis starts in the in the aorta-gonad-mesonephron area of the dorsal aorta of day 27–40 of human embryos [59] to generate multipotent, self-renewing HSCs from which the entire adult hematopoietic system will originate. In both cases, in vitro formation of mesoderm from PSCs, followed by induction of hemogenic endothelium (HE), is a prerequisite for subsequent hematopoietic specification (Figure 2a).

This occurs through an endothelial-to-hematopoietic transition that gives rise to CD45^+^ cells. In vitro establishment of primitive hematopoiesis has been demonstrated by different groups, with generation of RBCs mainly characterized by low enucleation potential, fetal globin expression and low expansion rate [60], and of macrophages resembling the embryonically-derived tissue-resident macrophages, rather that the BM monocyte-derived macrophages [61]. The activin-nodal pathway is required for primitive hematopoiesis specification, while specification of definitive hematopoiesis requires stage-specific activation of the canonical Wnt–β-catenin signaling, which in turn induces the expression of the caudal-related homeobox 4 (CDX4) transcription factor [62]. The expression of specific cell surface molecules and transcription factors at different stages during differentiation indicates the presence of mesoderm, HE and if primitive *vs* definitive hematopoiesis is being generated, with some differences in the choice of markers at each checkpoint from laboratory to laboratory. In addition to gene expression, the type of hematopoiesis is demonstrated through functional assays, for example, definitive but not primitive hematopoiesis gives rise to T cells in vitro. For regenerative medicine, the goal is to establish definitive hematopoiesis, with in vitro generation of HSCs able to give rise to all adult-type blood lineages (Table 1) including immunocompetent T and B lymphocytes for the treatment of immunodegenerative diseases. Generation of mature B and T cells in vitro from PSCs is particularly challenging. Differentiation of human iPSCs to immature B lymphocytes expressing surface IgM and undergoing VDJ recombination has been achieved [63]. However, even with improved protocols [64], at present it is possible to model in vitro only the earliest stages of human B lineage development occurring in utero. Immunocompetent B lymphopoiesis has been recently achieved in vivo in B-cell deficient mice after transplantation of ESCs transduced with three transcription factors (*Lhx2*, *Hoxa9*, and *Runx1*) essential to guide B-cell differentiation, hence upon further manipulation, likely without going through an in vitro PSC-derived HSC intermediate [65]. Likewise, engraftable human iPSC-derived hematopoietic progenitors with thymus-homing features and abundant TCRαβ repertoire was demonstrated upon transient expression of exogenous Runx1 and Hoxa9 in a specific time-window during in vitro differentiation [66], however mature T cells were generated only in vivo.

The gold-standard assay to demonstrate HSC functionality is the ability to repopulate long-term an irradiated or myeloablated murine host and to be serially transplanted. Generation of HSCs from human iPSCs fulfilling these criteria has been achieved successfully upon insertion of additional HSC-specific transcription factors [67], thus, adding further genetic manipulation of PSCs, not ideal for safety reasons and poor scalability. Moreover, although the quoted study certainly represents a fundamental milestone in the field, bona fide HSCs completed their development only in vivo in the xenograft model after intrabone rather than intravenous injection, indicating that the final phases of HSCs generation rely on in vivo niche factors, and that HSCs developing from PSCs might lack crucial homing signals. A reliable, efficient, fast, and clinically acceptable protocol for generating HSCs in vitro is still lacking, however considerable improvements have been achieved in recent years. For example, temporally controlled overexpression of critical regulators for mesodermal, endothelial, and/or hematopoietic specification (forward programming) has been proposed to overcome the difficulty in generating sufficient amounts of hemato-endothelial progenitors, often requiring purification from heterogeneous cell populations before hematopoietic specification [68]. We are confident that the continuous refinement and amelioration of current protocols will reach the goal of obtaining HSCs with long-term repopulating capacity. A brief overview of methods to generate HSPCs from PSCs is provided below; detailed protocols have been recently reviewed elsewhere [38,60,69,70]. Although developmental steps to generate primitive and definitive hematopoiesis are the same for mammals, timing and requirement of specific molecules and growth factors vary between mouse and human. Similarly, cell surface markers used to monitor each developmental step and to assess if definitive hematopoiesis is being obtained do not entirely overlap between mouse and human. We here mainly refer to methods for obtaining human hematopoiesis (Figure 2b).

### 4.1. Two-Dimensional (2D) In Vitro Differentiation

Primitive hematopoiesis can be generated from human PSCs via 2D monolayer systems in which sequential addition of specific cytokines direct mesoderm formation and subsequently differentiation to hematopoietic progenitor cells with erythroid, Mk and myeloid potential [71]. The necessary supportive stromal layer can either be iPSC-derived [71], or provided exogenously co-culturing differentiating iPSCs with OP9 cells or other cell lines, at early stages during mesoderm formation [72,73] or later to direct hematopoietic specification [74]. 2D systems can also be used to give rise to HSPCs more closely resembling definitive hematopoiesis, obtained for example after iPSC inducible forward programming [68], or by using scalable, clinically relevant commercial differentiation platforms, although not sufficient to generate engraftable HSCs [75]. As a further example, by seeding iPSC clumps or single cells in a vitronectin-coated surface, mesoderm induction occurs in two days in the presence of FGF2 and BMP4 [8]. This approach has been used with the purpose of generating adult-type Mk (Table 1), after patterning the definitive hematopoiesis program through the addition of Wnt signaling pathway agonists.

### 4.2. Three-Dimensional (3D) In Vitro Differentiation

Embryoid bodies (EBs) are 3D cell aggregates that recapitulate the spatial organization within the embryo and that allow the formation of endoderm, ectoderm, and mesoderm, the three germ layers. Addition of specific bone morphogenetic proteins and growth factors at the proper timing and concentration directs preferential formation of mesoderm over ectoderm or endoderm. To mimic primitive hematopoiesis, 3D-supported differentiation guarantees more efficient formation of mesoderm, commitment to hemogenic endothelium, generation of CD34^+^ hematopoietic cells, and colony-forming capacity compared to 2D [76], likely because the 3D EB system mimics the in vivo embryonic events. The EB system is used also for induction of definitive hematopoiesis, with proper stage-specific Activin inhibition, manipulation of the Wnt–β-catenin pathway, and correct choice of growth factors [77,78,79]. EBs can be generated in bulk cultures, in individual 96-well plate wells, with or without the hanging drop method. Composition of EB differentiation media, HE-inducing media, and of media for further hematopoietic differentiation into HSPC or mature cell type, as well as schedule of media exchanges and length of the different steps, vary from laboratory to laboratory and might result in different efficiencies if starting from established ESC lines or newly generated patient iPSCs; the time points at which the various cytokines are added might also need to be adjusted from line to line. In general, EB generation represents a delicate step and might be tricky if not carefully performed [79]. In addition, there is variability from donor to donor and even within each experiment using the same iPSC donor regarding the size and differentiation status of individual EBs, and often the operator discards suboptimal EBs before proceeding with the subsequent steps of differentiation. This represents a potential challenge for translation to a clinical-grade large-scale reproducible process.

An alternative 3D-culture HSPC differentiation method is based on favoring the emergence in vitro of sac-like structures, named ESC–derived sacs (ES-sacs), consisting in multiple cysts demarcated by cellular monolayers that concentrate hematopoietic progenitors. This method, initially used to demonstrate generation of functional platelets from ESCs [80], has been later adopted to generate HSPCs and RBCs from gene-edited human iPSCs [81]. However, similar to the EB method, reproducibility issues represent a challenge for future clinical application.

### 4.3. In Vivo Approaches

PSCs generate teratomas when transplanted in immunodeficient murine hosts. Teratomas recapitulate development of all body tissues and include a niche-like microenvironment permissive for HSC development [82]. Therefore, human iPSC-derived teratomas have been proposed as HSC source [83]. However, adaptation of teratoma-based protocols for large-scale HSC generation for clinical use is difficult to achieve.

## 5. Generation of Terminally Differentiated Hematopoietic Cells for Therapeutic Purposes

### 5.1. Platelets

In transfusion medicine a stable and controlled source for continuous production of platelets would be sufficient to treat thrombocytopenia due to inherited BM failure. Recent advances in differentiating Mk (from which platelets are released) from human PSCs and subsequent generation of platelets with improved output and safety paves the way for future clinical application [8]. The authors efficiently generated Mk from selected human iPSCs and ESCs through non-viral ectopic expression of specific transcription factors. They obtained large amounts of functional platelets using a scalable bioreactor system mirroring shear stress, and tailored lipid- and high glucose-supplemented culture media (Table 1).

### 5.2. Erythrocytes

An illimited source of universal donor RBCs would represent safer alternatives to conventional RBC units and solve issues related to scarce donor availability as has occurred during the COVID-19 pandemic. A major step forward in this direction has been achieved recently with a protocol involving reprogramming of ex-vivo expanded primary erythroblasts from human PB, mesoderm induction, differentiation to HSPC with a 2D commercial system, maturation to the erythroid lineage by addition of human platelet lysate (hPL) or human serum, and finally by in vitro expansion [84]. Interestingly, while only a small proportion of enucleated mature RBCs were present in culture, this fraction increased in vivo after transfusion in NSG immunodeficient mice pre-treated with macrophages and complement depleting agents. The authors showed that infused cells homed to the BM where they completed their differentiation and egressed into the PB. This holds promise for future clinical applications, since it can be foreseen that iPSC-derived RBCs would similarly complete their terminal differentiation within the human BM, once infused in patients. However, safety concerns associated with injection of nucleated RBC in patients must be addressed before this strategy can be translated into clinical practice.

Generating RBCs from gene-edited iPSCs would allow to overcome the occurrence of erythrocyte alloimmunization by recipients of repeated transfusions such as sickle cell anaemia and thalassemia patients. Bi-allelic correction of homozygous sickle cell mutations in patient-derived iPSCs and subsequent differentiation into adult-type erythrocytes has indeed been demonstrated, indicating the potential of this approach [81]. Similarly, talen-mediated gene edited pyruvate kinase deficiency patient-specific iPSCs were shown to give rise to healthy erythroid cells [85]. On the other hand, the problem of finding and recruiting donors with the suitable RBC phenotype on a constant basis would be solved by creating illimited sources of RBC with desired rare antigenic profiles [86]. Nevertheless, improvement of culture systems and of scale-up methods is still required for obtaining from PSCs the vast numbers of RBCs required in each unit of transfused blood.

### 5.3. Osteoclasts

Osteoclasts (OCs) are terminally differentiated, multinucleated cells derived from myeloid precursors, whose function is bone resorbtion. Together with osteoblasts, OCs are responsible for bone remodelling, which takes place throughout life and is crucial particularly during infancy and childhood. Defects in OC resorbing activity, such as those caused by mutations in the TCIRG1 gene, lead to ARO, a rare disease characterized by high bone mineral density and that can be cured with HSC transplantation from HLA-matched siblings. PSC-derived OCs might overcome the donor availability issue. In the absence of suitable transplantable HSCs, osteopetrotic infants could benefit from repeated infusions of OCs or of their progenitors, as proposed [87]. Functional adult-type osteoclasts have been recently obtained from human ESCs [88]. Importantly, iPSCs have been generated from *TCRG1*-mutated ARO patients [89]; once transduced with WT TCRG1, iPSCs from ARO subjects could be differentiated in vitro into bone resorbing OCs [90,91]. The novel osteopetrotic xenograft model that we recently generated might be used in future studies aimed at testing the in vivo relevance of human iPSCs-derived OCs [92]. 

## 6. Conclusions

In this review, we provided an overview of potential applications of PSCs for hereditary blood diseases and summarized different genome correction approaches and differentiation strategies towards to hematopoietic lineage. Regenerative medicine can greatly benefit from the iPSC technology, and we are confident that current and future research will address and partially solve the existent obstacles for this new tool to enter the clinical practice.

As mentioned, crucial issues that remain to be solved are proper differentiation to true HSCs, and the inherent donor-to-donor variability in responding to differentiation cues that affects the efficiency and quality of hematopoietic generation and of subsequent expansion. For large-scale production of platelets, RBCs or, in prospect, of HSCs from HLA-matched universal donors, prior testing of the most suitable cell line for each purpose can be easily envisioned. However, opting for classical BM transplantation from healthy donors or for using iPSCs from cell banks might be the best choice over transplant of corrected autologous iPSC-derived HSCs when these are not obtained in vitro in sufficient number or quality from individual patients. Further research to understand factors responsible for this variability is highly warranted before autologous iPSC-derived HSC transplantation become a reality in clinical settings.

## Figures and Tables

**Table 1:** Examples of generation of human adult-type differentiated hematopoietic cells in vitro from PSCs.

Final Cell Type	PSCs	Differentiation Protocol Steps	Validation	Ref.
RBCs	iPSCs from primary erythroblasts	1. STEMdiff to HSPCs 2. Ery expansion 3. hPL or plasma	FACS; transfusion in NSG mice pre-treated with CL and CVF	[84]
	MSC-iPS1	1. 3D mesoderm induction 2. Hematop. specification 3. Culture with OP9-DL1	BFU-E, analysis of globin gene expression	[77]
	OT1-1	1. 2D diff to HSPCs 2. Ery diff and expansion	FACS, morphology, enucleation	[86]
	iPSCs from sickle cell anemia pt dermal fibroblasts	1. 3D (ES sacs) 2. CD34^+^ isolation 3. Ery diff	FACS	[81]
PLT	PSCs from cell repositories	1. 2D mesoderm induction 2. Mk diff 3. Plt release in bioreactor	FACS; in vitro thrombus formation, transfusion in NRG/J mice	[8]
T and NK cells	MSC-iPS1	1. 3D mesoderm induction 2. 2D hematop specification 3. Culture with OP9-DL4	FACS	[77]
OCs	iPSCs from pt skin fibroblasts	1. 2D OP9 co-culture to HC 2. Myelo-prog. expansion 3. OC prog. expansion 4. OC maturation medium	TRAP staining, morphology, bone resorption	[90]
	iPSCs from pt skin fibroblasts	1. 3D mesoderm induction 2. monocyte diff medium 3. OC diff on bone slices	CTX-I release, TRAP staining, resorption	[91]

## Abbreviations

Pt	patient
Ery	erythroid
PL	platelets lysate
Hematop.	Hematopoietic
BFU-E	burst-forming unit-Ery
Diff	differentiation
Plt	platelets
HC	hematopoietic cells
Prog	progenitors
